# Impact of LDLR polymorphisms on lipid levels and atorvastatin’s efficacy in a northern Chinese adult Han cohort with dyslipidemia

**DOI:** 10.1186/s12944-024-02101-4

**Published:** 2024-04-14

**Authors:** Hong-Liang Zhao, Yang You, Yan Tian, Luyan Wang, Yongqiang An, Guoqiang Zhang, Chang Shu, Mingxin Yu, Yihua Zhu, Qian Li, Yanwei Zhang, Ningling Sun, Songnian Hu, Gang Liu

**Affiliations:** 1 Department of Cardiology, The First Hospital of Hebei Medical University, Shijiazhuang, Hebei China; 2 Beijing E-Seq Medical Technology Co. Ltd, Beijing, China; 3 State Key Laboratory of Microbial Resources, Institute of Microbiology, Chinese Academy of Sciences, Beijing, China; 4 College of Information Science and Technology, Nanjing Agricultural University, Nanjing, Jiangsu Province China; 5 Institute of Hypertension, People’s Hospital, Peking University, Beijing, China; 6 University of Chinese Academy of Sciences, Beijing, China

**Keywords:** Pharmacogenetics, LDLR polymorphisms, Atorvastatin, Dyslipidemia, Han Chinese

## Abstract

**Background:**

Dyslipidemia, a significant risk factor for atherosclerotic cardiovascular disease (ASCVD), is influenced by genetic variations, particularly those in the low-density lipoprotein receptor (LDLR) gene. This study aimed to elucidate the effects of LDLR polymorphisms on baseline serum lipid levels and the therapeutic efficacy of atorvastatin in an adult Han population in northern China with dyslipidemia.

**Methods:**

In this study, 255 Han Chinese adults receiving atorvastatin therapy were examined and followed up. The 3’ untranslated region (UTR) of the LDLR gene was sequenced to identify polymorphisms. The associations between gene polymorphisms and serum lipid levels, as well as changes in lipid levels after intervention, were evaluated using the Wilcoxon rank sum test, with a *P* < 0.05 indicating statistical significance. Assessment of linkage disequilibrium patterns and haplotype structures was conducted utilizing Haploview.

**Results:**

Eleven distinct polymorphisms at LDLR 3’ UTR were identified. Seven polymorphisms (rs1433099, rs14158, rs2738466, rs5742911, rs17249057, rs55971831, and rs568219285) were correlated with the baseline serum lipid levels (*P* < 0.05). In particular, four polymorphisms (rs14158, rs2738466, rs5742911, and rs17249057) were in strong linkage disequilibrium (r^2^ = 1), and patients with the AGGC haplotype had higher TC and LDL-C levels at baseline. Three polymorphisms (rs1433099, rs2738467, and rs7254521) were correlated with the therapeutic efficacy of atorvastatin (*P* < 0.05). Furthermore, carriers of the rs2738467 T allele demonstrated a significantly greater reduction in low-density lipoprotein cholesterol (LDL-C) levels post-atorvastatin treatment (*P* = 0.03), indicating a potentially crucial genetic influence on therapeutic outcomes. Two polymorphisms (rs751672818 and rs566918949) were neither correlated with the baseline serum lipid levels nor atorvastatin’s efficacy.

**Conclusions:**

This research outlined the complex genetic architecture surrounding LDLR 3’ UTR polymorphisms and their role in lipid metabolism and the response to atorvastatin treatment in adult Han Chinese patients with dyslipidemia, highlighting the importance of genetic profiling in enhancing tailored therapeutic strategies. Furthermore, this investigation advocates for the integration of genetic testing into the management of dyslipidemia, paving the way for customized therapeutic approaches that could significantly improve patient care.

**Trial registration:**

This multicenter study was approved by the Ethics Committee of Xiangya Hospital Central South University (ethics number K22144). It was a general ethic. In addition, this study was approved by The First Hospital of Hebei Medical University (ethics number 20220418).

**Supplementary Information:**

The online version contains supplementary material available at 10.1186/s12944-024-02101-4.

## Background

Dyslipidemia, characterized by abnormal lipid levels, emerges from complex interactions among genetics, lifestyle factors, metabolic stress, and autophagy [1–7]. Dyslipidemia is a major risk factor for atherosclerotic cardiovascular disease (ASCVD) which is the leading cause of death among Chinese urban and rural residents, and it accounts for more than 40% of deaths [8]. Epidemiological, genetic, and clinical intervention studies have identified low-density lipoprotein cholesterol (LDL-C) as a causal factor in ASCVD [9].

Statins, widely used to manage dyslipidemia, primarily mitigate ASCVD risk by effectively lowering LDL-C levels [10–12]. Despite widespread statin use, response varies due to multiple factors, including variations at the low-density lipoprotein receptor (LDLR) [13–15]. Numerous studies [16–39] have focused primarily on patients with familial hypercholesterolemia (FH) and have mostly examined coding regions and promoters of the LDLR gene. However, polymorphisms in the LDLR 3’ UTR have seldom been reported in the context of patients with dyslipidemia. A recent study revealed that variations in the LDLR 3’ UTR interfere with miRNA: mRNA interactions, which may impact gene expression and could be linked to FH [40].

This study investigated the impact of LDLR 3’ UTR polymorphisms on lipid levels before and after atorvastatin treatment in adult Chinese Han patients with dyslipidemia, offering significant insights into the genetic factors influencing serum lipid regulation and the potential effects on atorvastatin treatment outcomes. On one hand, this study provides an evidence for screening potential dyslipidemia population; on the other, it could help to identify the patients who benefit the most from taking atorvastatin, providing a strong guidance for clinical individualized precision treatment.

## Methods

### Study Population

This study enrolled 255 adult Chinese Han patients admitted to The First Hospital of Hebei Medical University between June 2022 and July 2023. All participants were prescribed a daily 20 mg dose of atorvastatin and underwent quarterly follow-up evaluations conducted by a skilled investigative team. Written informed consent confirming voluntary participation was obtained from each patient. This multicenter study was approved by the Ethics Committee of Xiangya Hospital Central South University (ethics number K22144). Ethical approval was also obtained from The First Hospital of Hebei Medical University (20220418).

### Data Collection

Baseline demographic characteristics, such as sex and age, were collected via interviews using a uniform questionnaire administered by trained researchers. Measurements of height and weight were taken at the nurse’s station by experienced nurses, and the body mass index (BMI) was determined by dividing the weight (in kilograms) by the square of the height (in meters).The blood of the participants was drawn from the antecubital vein in a fasting state by skilled nurses to measure triglyceride (TG), total cholesterol (TC), LDL-C, and high-density lipoprotein cholesterol (HDL-C) levels. All clinical investigations were conducted in accordance with the principles of the Declaration of Helsinki. At each follow-up, TG, TC, LDL-C and HDL-C levels were measured.

### DNA sequencing

From each enrolled patient, 2 ml of peripheral venous blood was collected for genomic DNA extraction using the Magnetic Blood Genomic DNA Kit (DP329, Tiangen Biotech Co., Ltd., Beijing, China). The DNA concentration was quantified with the Qubit® dsDNA HS Assay Kit (Yeasen Biotechnology Co., Ltd, Shanghai, China) according to the manufacturer’s protocol. The DNBSEQ-T7 sequencer (MGI Tech Co., Ltd, Shenzhen, China) was used for high-throughput sequencing of the DNA captured from a pharmacogenetics panel with reads of 150 bp in length.

### SNP calling and genotyping

High-quality sequencing reads were derived by filtering out adapters, unknown bases, and low-quality bases with Trimmomatic (v0.36) [41]. The high-quality reads were aligned to the human reference genome hg19 using the Burrows-Wheeler Aligner (BWA, v0.7.15) with the default parameters [42]. The Genome Analysis Toolkit (GATK, v3.8) was used for indel realignment, quality score recalibration, polymorphism calling, and genotyping (using Haplotype Caller) [43].

### Statistical analysis

Changes in serum lipid levels were quantified by calculating the difference from baseline to follow-up. The Δ%TG, Δ%TC, Δ%LDL-C, and Δ%HDL-C, represented the percentage changes in TG, TC, LDL-C and HDL-C, respectively. Associations between gene polymorphisms and serum lipid levels, including changes post-intervention, were evaluated with the Wilcoxon rank sum test. A *P* threshold of less than 0.05 indicated statistical significance. Assessment of linkage disequilibrium patterns and haplotype structures was conducted using Haploview software [44].

## Results

### Baseline characteristics of the study cohort

The baseline demographics of the 255 study participants are outlined in Table 1. The cohort predominantly comprised males (approximately 69%), and the majority of patients (over 78%) were aged between 50 and 80 years. A significant proportion of the patients (more than 70%) had a BMI greater than 24 kg/m².

**Table 1:** Baseline characteristics of the patients in this study

Characteristics		All patients (*n* = 255)
Sex	Male	177 (69.41%)
	Female	78 (30.59%)
Age, years	20 ∼ 29	2 (0.78%)
	30 ∼ 39	12 (4.71%)
	40 ∼ 49	27 (10.59%)
	50 ∼ 59	62 (24.31%)
	60 ∼ 69	75 (29.41%)
	70 ∼ 79	63 (24.71%)
	≥ 80	14 (5.49%)
BMI, kg/m^2^	< 18.5	3 (1.18%)
	18.5 ∼ 24	73 (28.63%)
	24 ∼ 28	120 (47.06%)
	≥ 28	59 (23.14%)

*Note* Values are presented as numbers (percentages)

### Distribution and frequency of LDLR polymorphisms

**Fig. 1:**
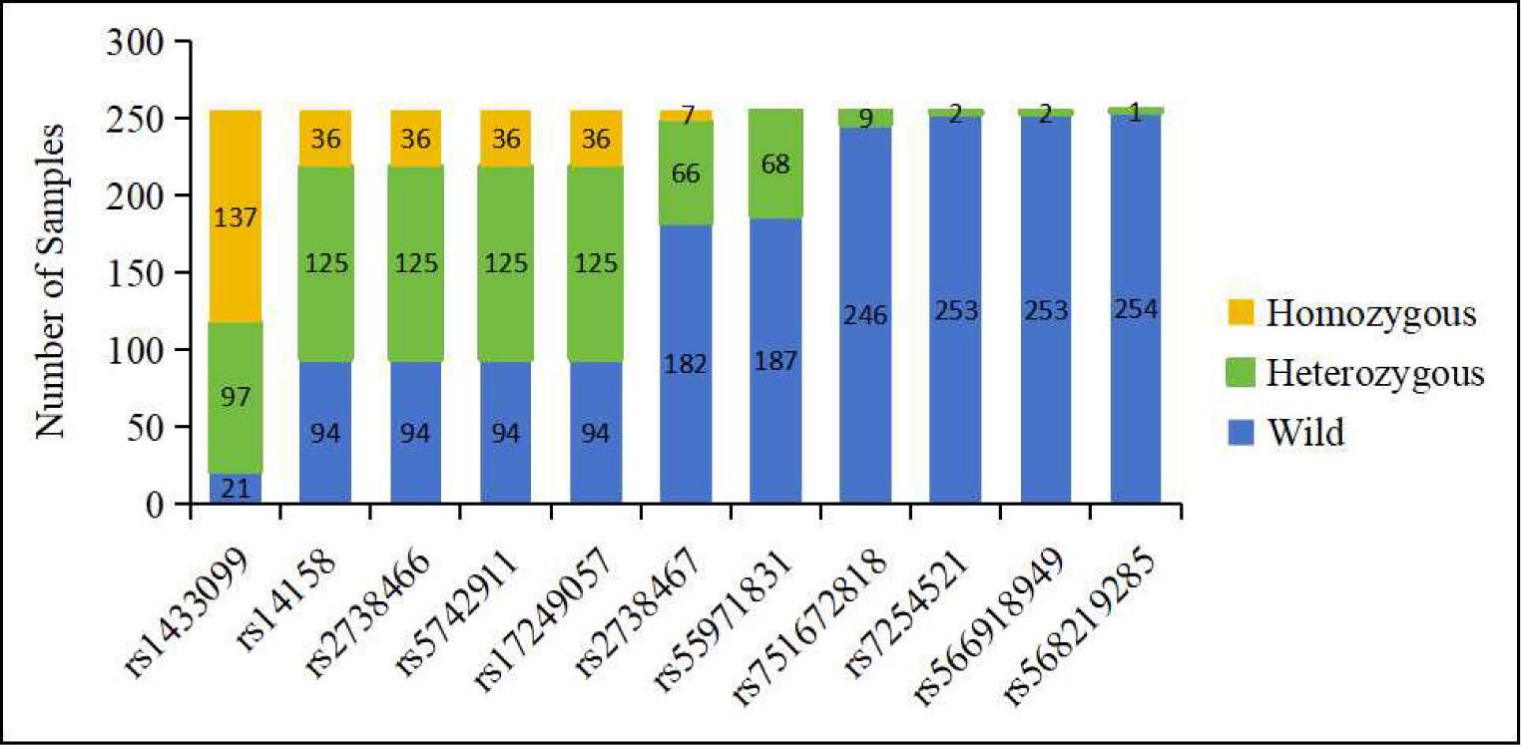
Polymorphisms in the LDLR 3’ UTR identified in this study

Eleven distinct LDLR polymorphisms within the 3’ UTR were identified across the study population, as detailed in Fig. 1 and Supplemental Table 1. The polymorphisms rs14158, rs2738466, rs5742911, and rs17249057 were identified concurrently in 255 patients, indicating an inheritance pattern. The genotype distribution for these four polymorphisms was that 94 patients (36.86%) were wild, 125 (49.02%) were heterozygous, and 36 (14.12%) were homozygous. The rs1433099 mutant allele was common, occurring in heterozygosity in 38.04% and in homozygosity in 53.73% of patients. The rs2738467 mutant allele was found in heterozygous form in 25.88% of patients and in homozygous form in 2.75% of patients. The rs55971831 mutant allele was present in 26.67% of patients, all of whom were heterozygous for the mutation. The rs751672818 mutant allele occurred in 3.53% of patients, exclusively in heterozygous form. The mutant alleles of rs568219285, rs7254521, and rs566918949 were rare, being detected in only one or two individuals.

The identified polymorphisms, especially those exhibiting multiple genotype occurrences, warrant further investigation as potential markers for dyslipidemia in the Chinese population.

### Comparison of Allele frequencies to those in public databases

**Fig. 2:**
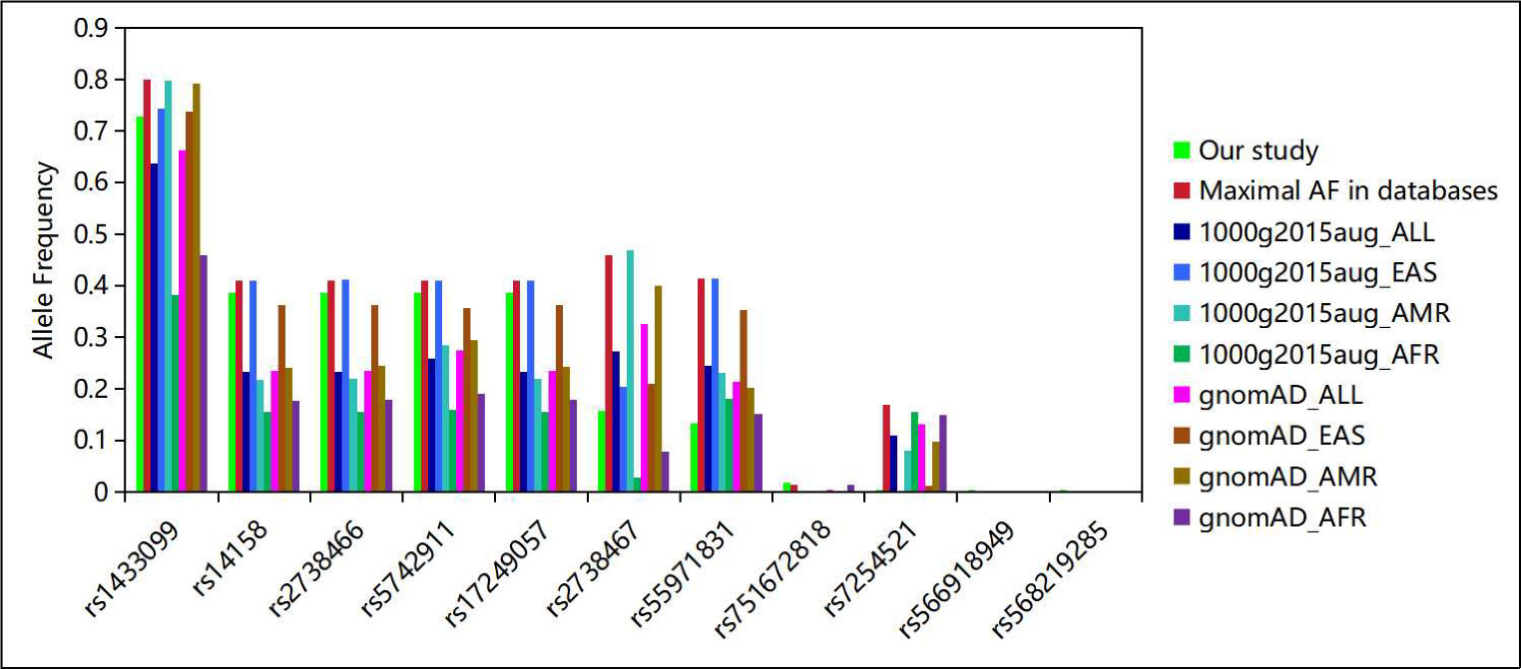
The AFs of the identified polymorphisms in this study and public databases

The allele frequencies (AFs) of the identified polymorphisms were compared with those reported in public genomic databases, as detailed in Fig. 2 and Supplemental Table 2. Except for rs55971831, the AFs of the other ten identified polymorphisms closely matched those observed in East Asian populations within the August 2015 release of the 1000 Genomes Project (1000g2015aug) and the Genome Aggregation Database (gnomAD). The AF for rs55971831 was 0.13 in this cohort, lower than that reported for East Asian populations in both 1000g2015aug and gnomAD. The AFs for rs14158, rs2738466, rs5742911, and rs17249057 were 0.39 in this study. They were slightly lower than the highest recorded AF of 0.41 in East Asian populations, but significantly higher than the AFs observed in American (ranging from 0.21 to 0.29) and African populations (ranging from 0.15 to 0.19). This disparity in AFs suggests a genetic predisposition within the Chinese population for these specific LDLR polymorphisms, underscoring their potential as markers of dyslipidemia in this ethnic group. The AF of rs1433099 was observed to be 0.73 in this study. In contrast, in the 1000g2015aug and gnomAD databases, the AF was reported at 0.79 in American populations, and it ranged between 0.38 and 0.46 in African populations. This indicates that rs1433099 is a common polymorphism across different ethnicities. The AF for rs2738467 was 0.16 in this study, and it was 0.40 to 0.47 in American populations and 0.03 to 0.08 in African populations. This significant variation indicates that the rs2738467 polymorphism exhibits considerable diversity in different populations. The AF of rs7254521 was 0.004 in this study, and this value was 0.003 ∼ 0.132 in the East Asian population in the public database. However, the AF of rs7254521 was 0.08 in the American population, and approximately 0.15 in the African population. This indicates that rs7254521 has a high ethnic diversity. The polymorphisms rs751672818, rs566918949, and rs568219285 exhibited low AFs in all populations studied, each being less than 0.02. This suggests that these are rare polymorphisms.

1000g2015aug: August 2015 release of the 1000 Genomes Project, gnomAD: Genome Aggregation Database, All: All populations, EAS: East Asian, AMR: American, AFR: African.

### Linkage disequilibrium and Haplotype Analysis

**Fig. 3:**
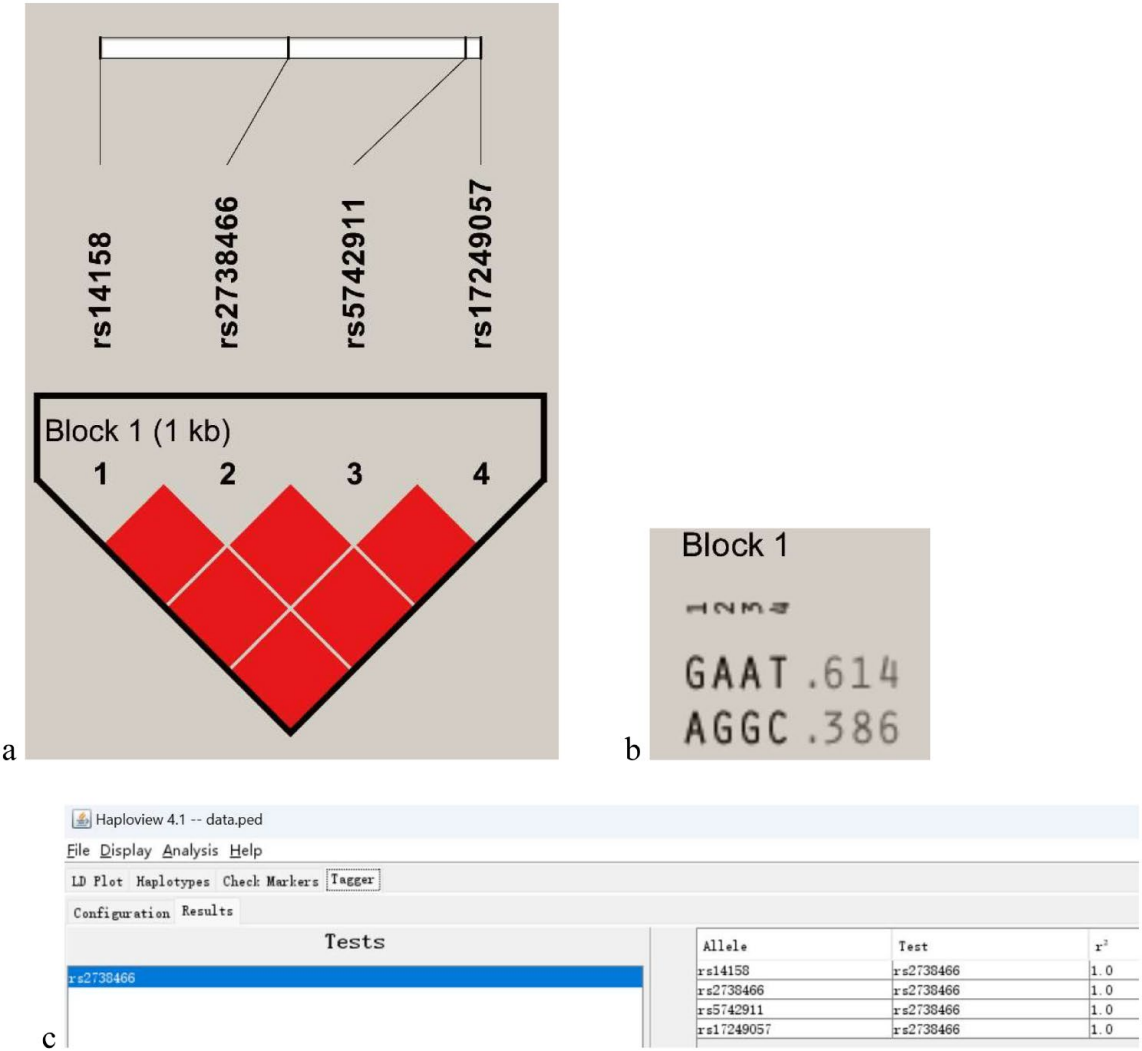
Linkage disequilibrium of LDLR polymorphisms and haplotypes. (**a**) Linkage disequilibrium (LD) plot generated using Haploview; (**b**) The haplotypes and frequencies for the identified block; (**c**) The evaluation of tag SNPs and corresponding statistical tests

The polymorphisms rs14158, rs2738466, rs5742911, and rs17249057, cooccurring in patients, were subjected to linkage disequilibrium analysis. The results, depicted in Fig. 3, revealed strong linkage disequilibrium among these polymorphisms (r^2^ = 1). The identified haplotypes, GAAT and AGGC, had population allele frequencies of 0.614 and 0.386, respectively, in this study cohort.

### Impact of LDLR polymorphisms on serum lipid levels at Enrollment

The impact of identified polymorphisms on serum lipid levels at enrollment was assessed, with findings summarized in Table 2. Significant associations were observed between polymorphisms rs14158, rs2738466, rs5742911, and rs17249057 and baseline levels of TC and LDL-C (*P* < 0.05). Individuals carrying the A allele of rs14158, the G allele of rs2738466, the G allele of rs5742911, and the C allele of rs17249057 displayed elevated TC and LDL-C levels compared to carriers of alternative alleles. This indicates that such polymorphisms, especially when inherited as a haplotype, could impact LDL-C metabolism. A recent study [40] showed that rs5742911 enhances or creates a binding site for three miRNAs (miR-3190-5p, miR-4435, and miR-4717-5p) and disrupts a binding site for miR-1587-5p, influencing gene expression and potentially contributing to FH, underscoring the significance of the findings in this study. Polymorphism rs1433099 was strongly associated with baseline TC and LDL-C levels (*P* < 0.05). Those who carry the C allele had higher levels of TC and LDL-C. Polymorphism rs55971831 was significantly associated with TG levels (*P =* 0.002), carriers of the A allele exhibiting higher TG levels than those with the C allele. Polymorphism rs568219285 exhibited a significant correlation with baseline TG and TC levels (*P* < 0.05). However, due to its rarity, further validation in a larger cohort is necessary. No significant correlations were observed between polymorphisms rs2738467, rs751672818, rs7254521, or rs566918949 and baseline serum lipid levels.

### Influence of of LDLR polymorphisms on Atorvastatin Treatment Efficacy

**Fig. 4:**
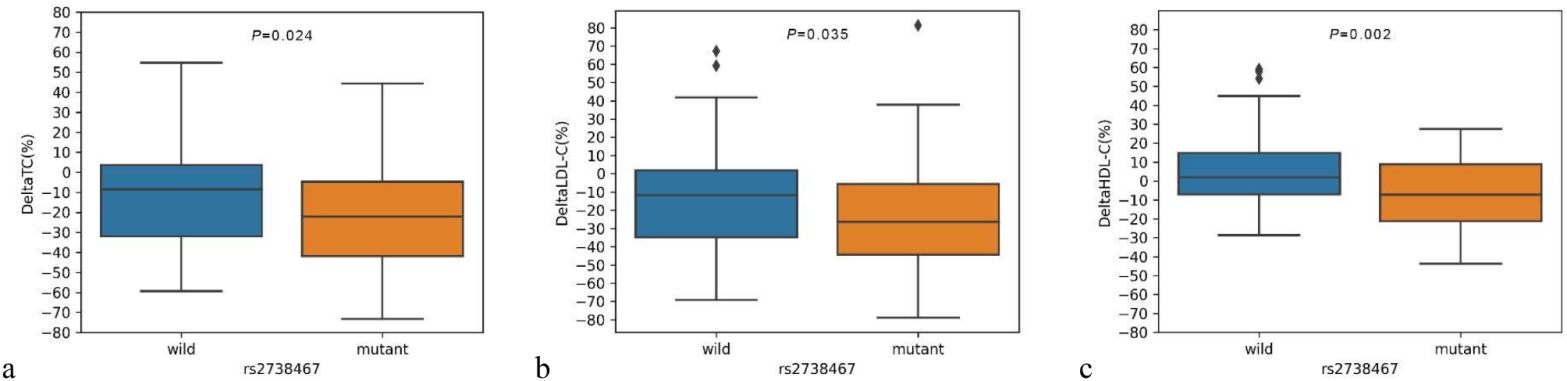
The relative changes in lipid levels in patients with different genotypes at locus rs2738467 after atorvastatin therapy. (**a**) DeltaTC (%) = 100*(TCpostintervention-TCenrollment)/TCenrollment; (**b**) DeltaLDL-C (%) = 100*(LDL-Cpostintervention-LDL-Cenrollment)/LDL-Cenrollment; (**c**) DeltaHDL-C (%) = 100*(HDL-Cpostintervention-HDL-Cenrollment)/HDL-Cenrollment

The relationship between LDLR polymorphisms and the relative change in serum lipid levels after atorvastatin therapy was evaluated and was showed in Table 3. Participants carrying the rs2738467 T allele showed a more significant reduction in TC, LDL-C, and HDL-C levels than did those with the C allele (*P* < 0.05). This novel discovery suggests that the rs2738467 T allele might augment the cholesterol-lowering efficacy of atorvastatin. The relative changes in lipid levels in patients with different genotypes at locus rs2738467 after atorvastatin therapy were shown in Fig. 4. TC and LDL-C levels reduced 20% in patients carrying the rs2738467 T allele and 10% in those with the C allele. Although HDL-C levels also decreased in patients with the rs2738467 T allele, the median change was under 5%, with some patients even experiencing an increase in HDL-C levels. This suggests that the rs2738467 T allele may specifically enhance atorvastatin’s efficacy in lowering LDL-C levels. The rs1433099 showed a significant correlation with change in HDL-C levels post-atorvastatin treatment (*P* = 0.02). Although patients carrying the rs1433099 C allele presented with greater TC and LDL-C levels at baseline, they showed a greater improvement in HDL-C levels following atorvastatin treatment. The rs7254521 was strongly associated with LDL-C levels post-atorvastatin treatment (*P* = 0.03); however, this observation was limited to only two patients. Verification in larger cohorts is necessary in future studies. No significant correlations were observed between polymorphisms rs14158, rs2738466, rs5742911, rs17249057, rs55971831, rs751672818, rs566918949, or rs568219285 and atorvastatin’s efficacy.

**Table 2:** The correlation between LDLR polymorphisms and serum lipid levels at enrollment

rsID	gDNA_coordinate	wild/mutant (patients)	P							
			TG (wild greater)	TG (mutant greater)	TC (wild greater)	TC (mutant greater)	LDL-C (wild greater)	LDL-C (mutant greater)	HDL-C (wild greater)	HDL-C (mutant greater)
rs1433099	chr19:g.11242658T > C	21/234	0.807	0.193	0.977	**0.023**	0.981	**0.019**	0.517	0.483
rs14158	chr19:g.11242044G > A	94/161	0.686	0.314	0.995	**0.005**	0.992	**0.008**	0.844	0.156
rs2738466	chr19:g.11,242,765 A > G	94/161	0.686	0.314	0.995	**0.005**	0.992	**0.008**	0.844	0.156
rs5742911	chr19:g.11,243,445 A > G	94/161	0.686	0.314	0.995	**0.005**	0.992	**0.008**	0.844	0.156
rs17249057	chr19:g.11243502T > C	94/161	0.686	0.314	0.995	**0.005**	0.992	**0.008**	0.844	0.156
rs2738467	chr19:g.11,243,735 C > T	182/73	0.467	0.533	0.401	0.599	0.509	0.491	0.727	0.273
rs55971831	chr19:g.11,243,411 C > A	188/67	0.998	**0.002**	0.386	0.614	0.264	0.736	0.025	0.975
rs751672818	chr19:g.11243411delC	246/9	0.181	0.819	0.331	0.669	0.333	0.667	0.658	0.342
rs7254521	chr19:g.11,243,422 C > T	253/2	0.17	0.83	0.231	0.769	0.165	0.835	0.847	0.153
rs566918949	chr19:g.11243467G > A	253/2	0.422	0.578	0.692	0.308	0.803	0.197	0.401	0.599
rs568219285	chr19:g.11242719G > A	254/1	0.958	**0.042**	0.955	**0.045**	0.514	0.486	0.061	0.939

*Note* Serum lipid levels at enrollment were compared by the Wilcoxon rank sum test. Values in bold are statistically significant (*P* < 0.05). The *P* values listed in the table represent the null hypothesis, while the remarks in parentheses are indicative of the alternative hypothesis

**Table 3:** Associations between LDLR polymorphisms and the percentage changes in serum lipid levels after atorvastatin therapy

rsID	gDNA_coordinate	wild/mutant (patients)	P							
			Δ%TG (wild greater)	Δ%TG (mutant greater)	Δ%TC (wild greater)	Δ%TC (mutant greater)	Δ%LDL-C (wild greater)	Δ%LDL-C (mutant greater)	Δ%HDL-C (wild greater)	Δ%HDL-C (mutant greater)
rs1433099	chr19:g.11242658T > C	21/234	0.461	0.539	0.571	0.429	0.514	0.486	**0.02**	0.98
rs14158	chr19:g.11242044G > A	94/161	0.707	0.293	0.876	0.124	0.851	0.149	0.639	0.361
rs2738466	chr19:g.11,242,765 A > G	94/161	0.707	0.293	0.876	0.124	0.851	0.149	0.639	0.361
rs5742911	chr19:g.11,243,445 A > G	94/161	0.707	0.293	0.876	0.124	0.851	0.149	0.639	0.361
rs17249057	chr19:g.11243502T > C	94/161	0.707	0.293	0.876	0.124	0.851	0.149	0.639	0.361
rs2738467	chr19:g.11,243,735 C > T	182/73	0.309	0.691	**0.024**	0.976	**0.035**	0.965	**0.002**	0.998
rs55971831	chr19:g.11,243,411 C > A	188/67	0.176	0.824	0.712	0.288	0.862	0.138	0.496	0.504
rs751672818	chr19:g.11243411delC	246/9	0.645	0.355	0.676	0.324	0.337	0.663	0.984	0.016
rs7254521	chr19:g.11,243,422 C > T	253/2	0.5	0.5	0.818	0.182	0.969	**0.031**	0.682	0.318
rs566918949	chr19:g.11243467G > A	253/2	0.286	0.714	0.238	0.762	0.261	0.739	0.122	0.878
rs568219285	chr19:g.11242719G > A	254/1	0.05	0.95	0.157	0.843	0.876	0.124	0.942	0.058

*Note* The relative changes in serum lipid levels after atorvastatin therapy were compared by the Wilcoxon rank sum test. Values in bold are statistically significant (*P* < 0.05). The *P* values listed in the table represent the null hypothesis, while the remarks in parentheses are indicative of the alternative hypothesis. Δ%TG = 100*(TGpostintervention-TGenrollment)/TGenrollment; Δ%TC = 100*(TCpostintervention-TCenrollment/TCenrollment; Δ%LDL-C = 100*(LDL-Cpostintervention-LDL-Cenrollment/LDL-Cenrollment; Δ%HDL-C = 100*(HDL-Cpostintervention-HDL-Cenrollment/HDL-Cenrollment.

## Discussion

This study not only provided a comprehensive analysis of the correlation between polymorphisms in the LDLR 3’ UTR and baseline serum lipid levels, but also revealed an association between these polymorphisms and the therapeutic efficacy of atorvastatin in a cohort of adult Chinese Han patients with dyslipidemia. The identification of 11 polymorphisms in the LDLR 3’ UTR of these patients underscored the genetic diversity within this population and highlighted the potential of these polymorphisms to serve as biomarkers for the treatment of dyslipidemia.

The polymorphisms rs14158, rs2738466, rs5742911, and rs17249057 which were in strong linkage disequilibrium, were significantly correlated with baseline serum lipid levels. Patients with the AGGC haplotype had higher LDL-C levels at baseline. Although an investigation within a southern Chinese population has not established a correlation between polymorphisms rs14158 and rs2738466 and the incidence of coronary heart disease [45], data from a black South African cohort indicated that carriers of the rs14158 A allele have elevated LDL-C levels, increasing the risk for FH [46]. In addition, research conducted in a Spanish population revealed that subjects with hypercholesterolemia harboring the rs14158 A allele and the rs2738466 G allele exhibit a diminished response to the lipid-modulating agent Armolipid Plus, suggesting that these specific SNPs may exacerbate hypercholesterolemia susceptibility [47]. Furthermore, according to a Mexican study, the rs14158 A allele and the rs2738466 G allele were associated with an increased risk of acute coronary syndrome and concomitantly lower HDL-C levels [48]. Additionally, rs5742911 was potentially associated with FH by disrupting interactions with miRNAs and altering gene expression in a recent Dutch study [40]. Collectively, these findings underscore the potential for the rs14158, rs2738466, rs5742911, and rs17249057 polymorphisms to influence cholesterol metabolism in various ways between distinct populations.

In this study, polymorphisms rs14158, rs2738466, rs5742911, and rs17249057 were not correlated with the therapeutic efficacy of atorvastatin. This finding was consistent with a study in Brazilian cohorts in which the allelic polymorphism rs14158G had no discernible influence on the therapeutic efficacy of atorvastatin [49]. However, a study in the United States showed that rs5742911 was associated with poor simvastatin response in black patients but not in white patients [50].

The rs2738467 T allele was associated with a more pronounced reduction in LDL-C levels after atorvastatin therapy but was not associated with baseline lipid levels. This finding suggests a potential role for this polymorphism in improving the efficacy of atorvastatin. This finding supports the precision medicine approach, which emphasizes customizing treatment plans according to individual genetic profiles.

The allele frequencies of the identified polymorphisms in this study were consistent with them in East Asian populations as documented in public genomic databases. This reinforces the validity of the findings and suggests a genetic predisposition among the Chinese population to these specific LDLR polymorphisms. The findings in this study have profound implications for population-specific genetic screening and therapeutic interventions.

### Study strengths and limitations

This research presents several strengths, notably its investigation into the effects of LDLR 3’ UTR polymorphisms on lipid levels both pre- and post-atorvastatin therapy in a population of adult Chinese Han individuals with dyslipidemia. The study provides valuable insights into the genetic factors that regulate serum lipids and how the factors impact the efficacy of atorvastatin treatment. This study not only supports the stratification of potential dyslipidemia cases for targeted screening but also aids in pinpointing individuals most likely to benefit from atorvastatin therapy. As a result, this work lays a foundation for the implementation of personalized, precision medicine in clinical settings.

This study still has several limitations. Firstly, focusing exclusively on adult Chinese Han patients with dyslipidemia might restrict the applicability of the findings to other ethnicities or demographics. Secondly, the infrequent presence of certain polymorphisms, like rs568219285, necessitates further exploration in more extensive and varied populations to verify their links to lipid profiles and medication effects. Lastly, while this study concentrated on the relationship between LDLR polymorphisms and lipid levels alterations post-atorvastatin treatment, other contributory factors and underlying mechanisms remain unexamined.

## Conclusions

In conclusion, this investigation has uncovered a significant link between LDLR gene 3’ UTR polymorphisms and lipid levels, as well as their impact on atorvastatin response. These insights open new pathways for advanced studies and clinical applications, highlighting the importance of genetic profiling in tailoring treatment for dyslipidemia. By adopting a personalized approach to therapy, it can enhance treatment precision and effectiveness, ultimately alleviating the cardiovascular disease burden associated with dyslipidemia.

### Electronic supplementary material

Below is the link to the electronic supplementary material.

Supplementary Material 1

Supplementary Material 2

Supplementary Material 3

Supplementary Material 4

## Data Availability

The datasets featured in this article are not openly accessible due to restrictions on the public dissemination of genomic information imposed by the Institutional Ethics Committee. To access the datasets, requests should be made to the corresponding authors.

## Abbreviations

ASCVD: Atherosclerotic cardiovascular disease
LDLR: Low-density lipoprotein receptor
FH: Familial hypercholesterolemia
UTR: Untranslated regions
TG: Triglyceride
TC: Total cholesterol
LDL-C: Low-density lipoprotein cholesterol
HDL-C: High-density lipoprotein cholesterol
