# Supplementary material for: Impact of LDLR polymorphisms on lipid levels and atorvastatin’s efficacy in a northern Chinese adult Han cohort with dyslipidemia
Source: Lipids Health Dis. 2024 Apr 14;23:106. doi: 10.1186/s12944-024-02101-4 (PMC11016223; doi:10.1186/s12944-024-02101-4)
Supplement: Supplementary file 3 — Supplementary Material 3 [file 12944_2024_2101_MOESM3_ESM.html]

# iThenticate

Document Viewer

Similarity Index

6%

## Impact of LDLR Polymorphisms on Lipid Levels an...

### By: Yan Tian

As of: Mar 29, 2024 1:55:44 AM
  
5,516 words - 18 matches - 8 sources

UndoInfo

# sources:

67 words / 1%
- Internet from 22-Feb-2022 12:00AM

www.dovepress.com

29 words / 1%
- from 20-Feb-2024 12:00AM

www.researchsquare.com

25 words / 1%
- Internet from 30-Jul-2020 12:00AM

www.researchsquare.com

45 words / 1%
- from 07-Nov-2023 12:00AM

lipidworld.biomedcentral.com

42 words / 1%
- from 20-Mar-2024 12:00AM

www.frontiersin.org

28 words / 1%
- from 16-Nov-2023 12:00AM

www.mdpi.com

25 words / 1%
- Crossref Posted Content

Kaihan Wang, Tingting Hu, Mengmeng Tai, Yan Shen, Haocheng Chai, Shaoyi Lin, Xiaomin Chen. "LDLR c.415G&gt;A causes familial hypercholesterolemia by weakening LDLR binding to LDL", Research Square Platform LLC, 2024

25 words / 1%
- Crossref

Renata Caroline Costa de Freitas, Raul Hernandes Bortolin, Jessica Bassani Borges, Victor Fernandes de Oliveira et al. "LDLR and PCSK9 3´UTR variants and their putative effects on microRNA molecular interactions in familial hypercholesterolemia: a computational approach", Molecular Biology Reports, 2023

# paper text:

Impact of LDLR Polymorphisms on Lipid Levels and Atorvastatin’s Efficacy in a Northern Chinese Adult Han Cohort with Dyslipidemia Hong-Liang Zhao1\*, Yang You1\*, Yan Tian2\*, Luyan Wang5, Yongqiang An1, Guoqiang Zhang2, Chang Shu3, Mingxin Yu2, Yihua Zhu2,4, Qian Li2, Yanwei Zhang2, Ningling Sun5†, Songnian Hu3,6†, Gang Liu1† 1 Department of Cardiology, The First Hospital of Hebei Medical University, Shijiazhuang, Hebei, China; 2 Beijing E-Seq Medical Technology Co. Ltd., Beijing, China; 3 State Key Laboratory of Microbial Resources, Institute of Microbiology, Chinese Academy of Sciences, Beijing, China; 4 College of Information Science and Technology, Nanjing Agricultural University, Nanjing, Jiangsu Province, China; 5 Institute of Hypertension, People’s Hospital, Peking University, Beijing, China; 6 University of Chinese Academy of Sciences, Beijing, China. Gang Liu https://orcid.org/0000-0003-1221-3698 Hong-Liang Zhao https://orcid.org/0000-0002-1331-8515 \*These authors contributed equally and are co-first authors. †These authors contributed equally and are corresponding authors. Correspondence: Gang Liu (cardio2004@hebmu.edu.cn), Songnian Hu (husn@im.ac.cn), Ningling Sun (sunnl@263.net) Abstract Background Dyslipidemia, a significant risk factor for atherosclerotic cardiovascular disease (ASCVD), is influenced by genetic variations, particularly those in the low-density lipoprotein receptor (LDLR) gene. This study aimed to elucidate the effects of LDLR polymorphisms on baseline serum lipid levels and the therapeutic efficacy of atorvastatin in an adult Han population in northern China with dyslipidemia. Methods In this study, 255 Han Chinese adults receiving atorvastatin therapy were examined and followed up. The 3’ untranslated region (UTR) of the LDLR gene was sequenced to identify polymorphisms. The associations between gene polymorphisms and serum lipid levels, as well as changes in lipid levels after intervention, were evaluated using the Wilcoxon rank sum test, with a P<0.05 indicating statistical significance. Assessment of linkage disequilibrium patterns and haplotype structures was conducted utilizing Haploview. Results Eleven distinct polymorphisms at LDLR 3’ UTR were identified. Seven polymorphisms (rs1433099, rs14158, rs2738466, rs5742911, rs17249057, rs55971831, and rs568219285) were correlated with the baseline serum lipid levels (P<0.05). In particular, four polymorphisms (rs14158, rs2738466, rs5742911, and rs17249057) were in strong linkage disequilibrium (r2=1), and patients with the AGGC haplotype had higher TC and LDL-C levels at baseline. Three polymorphisms (rs1433099, rs2738467, and rs7254521) were correlated with the therapeutic efficacy of atorvastatin (P<0.05). Furthermore, carriers of the rs2738467 T allele demonstrated a significantly greater reduction in low-density lipoprotein cholesterol (LDL-C) levels post-atorvastatin treatment (P=0.03), indicating a potentially crucial genetic influence on therapeutic outcomes. Two polymorphisms (rs751672818 and rs566918949) were neither correlated with the baseline serum lipid levels nor atorvastatin’s efficacy. Conclusions This research outlined the complex genetic architecture surrounding LDLR 3’ UTR polymorphisms and their role in lipid metabolism and the response to atorvastatin treatment in adult Han Chinese patients with dyslipidemia, highlighting the importance of genetic profiling in enhancing tailored therapeutic strategies. Furthermore, this investigation advocates for the integration of genetic testing into the management of dyslipidemia, paving the way for customized therapeutic approaches that could significantly improve patient care. Keywords Pharmacogenetics, LDLR polymorphisms, Atorvastatin, Dyslipidemia, Han Chinese Trial registration This multicenter

study was approved by the Ethics Committee of Xiangya Hospital Central South University (ethics

number K22144). It was a general ethic. In addition, 3 / 30 this study was approved by The First Hospital of Hebei Medical University (ethics number 20220418). Background Dyslipidemia, characterized by abnormal lipid levels, emerges from complex interactions among genetics, lifestyle factors, metabolic stress, and autophagy [1-7]. Dyslipidemia is a major risk factor for atherosclerotic cardiovascular disease (ASCVD) which is the leading cause of death among Chinese urban and

rural residents
, and it accounts
for more than 40% of deaths

[8].

Epidemiological, genetic, and clinical
intervention
studies
have identified
low-density lipoprotein cholesterol (LDL-C
) as
a causal factor
in
ASCVD

[9]. Statins, widely used to manage dyslipidemia, primarily mitigate ASCVD risk by effectively lowering LDL-C levels [10-12]. Despite widespread statin use, response varies due to multiple factors, including variations at the low-density lipoprotein receptor (LDLR) [13-15]. Numerous studies [16-39] have focused primarily on patients with familial hypercholesterolemia (FH) and have mostly examined coding regions and promoters of the LDLR gene. However, polymorphisms in the LDLR 3’ UTR have seldom been reported in the context of patients with dyslipidemia. A recent study revealed that variations in the LDLR 3’ UTR interfere with miRNA: mRNA interactions, which may impact gene expression and could be linked to FH [40]. This study investigated the impact of LDLR 3’ UTR polymorphisms on lipid levels before and after atorvastatin treatment in adult Chinese Han patients with dyslipidemia, offering significant insights into the genetic factors influencing serum lipid regulation and the potential effects on atorvastatin treatment outcomes. On one hand, this study provides an evidence for screening potential dyslipidemia population; on the other, it could help to identify the patients who benefit the most from taking atorvastatin, providing a strong guidance for clinical individualized precision treatment. Methods Study Population This study enrolled 255 adult Chinese Han patients admitted to The First Hospital of Hebei Medical University between June 2022 and July 2023. All participants were prescribed a daily 20 mg dose of atorvastatin and underwent quarterly follow-up evaluations conducted by a skilled investigative team. Written informed consent confirming voluntary participation was obtained from each patient. This multicenter

study was approved by the Ethics Committee of Xiangya Hospital Central South University (ethics
number K22144). Ethical
approval

was also obtained from The First Hospital of Hebei Medical University (20220418). Data Collection Baseline demographic characteristics, such as sex and age, were collected via interviews using a uniform questionnaire administered by trained researchers. Measurements of height and weight were taken at the nurse's station by experienced nurses, and the body mass index (BMI) was determined by dividing the weight (in kilograms) by the square of the height (in meters).The blood of the participants was drawn from the antecubital vein in a fasting state by skilled nurses to measure triglyceride (

TG), total cholesterol (TC), LDL-C
, and
high-density lipoprotein cholesterol (HDL-C) levels
. All clinical investigations
were
conducted in accordance
with

the principles of the Declaration of Helsinki. At each follow-up, TG, TC, LDL-C and HDL-C levels were measured. DNA Sequencing From each enrolled patient, 2 ml of peripheral venous blood was collected for genomic DNA extraction using the Magnetic Blood Genomic DNA Kit (DP329, Tiangen Biotech Co., Ltd., Beijing, China). The DNA concentration was quantified

with the Qubit® dsDNA HS Assay Kit (Yeasen
Biotechnology Co., Ltd,
Shanghai, China

) according to the manufacturer's protocol. The DNBSEQ-T7 sequencer (MGI Tech Co., Ltd, Shenzhen, China) was used for high-throughput sequencing of the DNA captured from a pharmacogenetics panel with reads of 150 bp in length.

SNP Calling and Genotyping High-quality sequencing reads were

derived by filtering out adapters, unknown bases, and low-quality bases with

Trimmomatic (v0.36
) [41].
The high-quality reads were aligned to
the
human reference genome hg19 using the Burrows-Wheeler Aligner (BWA, v0.7.15
) with
the default parameters
[42].
The Genome Analysis Toolkit (GATK, v3.8) was used for
indel
realignment, quality score recalibration
, polymorphism
calling, and genotyping (using Haplotype Caller
) [43].
Statistical Analysis

6 / 30 Changes in serum lipid levels were quantified by calculating the difference from baseline to follow-up. The Δ%TG, Δ%TC, Δ%LDL-C, and Δ%HDL-C, represented the percentage changes in TG, TC, LDL-C and HDL-C, respectively. Associations between gene polymorphisms and serum lipid levels, including changes post- intervention, were evaluated with the Wilcoxon rank sum test. A P threshold of less than 0.05 indicated statistical significance. Assessment of linkage disequilibrium patterns and haplotype structures was conducted using Haploview software [44]. Results Baseline

Characteristics of the Study
Cohort
The
baseline demographics
of the
255
study
participants
are
outlined
in Table 1. The

cohort predominantly comprised males (approximately 69%), and the majority of patients (over 78%) were aged between 50 and 80 years. A significant proportion of the patients (more than 70%) had a BMI greater than 24 kg/m². Table 1 Baseline characteristics of the patients in this study Characteristics All patients (n = 255) Sex Male 177 (69.41%) Female 78 (30.59%) Age, years 20~29 2 (0.78%) 30~39 12 (4.71%) 40~49 27 (10.59%) 50~59 62 (24.31%) 60~69 75 (29.41%) 70~79 63 (24.71%) >=80 14 (5.49%) BMI, kg/m2 <18.5 3 (1.18%) 18.5~24 73 (28.63%) 24~28 120 (47.06%) >=28 59 (23.14%) Note: Values are presented as numbers (percentages). Distribution and Frequency of LDLR Polymorphisms Eleven distinct LDLR polymorphisms within the 3’ UTR were identified across the study population, as detailed in Figure 1 and Supplemental Table 1. The polymorphisms rs14158, rs2738466, rs5742911, and rs17249057 were identified concurrently in 255 patients, indicating an inheritance pattern. The genotype distribution for these four polymorphisms was that 94 patients (36.86%) were wild, 125 (49.02%) were heterozygous, and 36 (14.12%) were homozygous. The rs1433099 mutant allele was common, occurring in heterozygosity in 38.04% and in homozygosity in 53.73% of patients. The rs2738467 mutant allele was found in heterozygous form in 25.88% of patients and in homozygous form in 2.75% of patients. The rs55971831 mutant allele was present in 26.67% of patients, all of whom were heterozygous for the mutation. The rs751672818 mutant allele occurred in 3.53% of patients, exclusively in heterozygous form. The mutant alleles of rs568219285, rs7254521, and rs566918949 were rare, being detected in only one or two individuals. The identified polymorphisms, especially those exhibiting multiple genotype occurrences, warrant further investigation as potential markers for dyslipidemia in the Chinese population. Figure 1 Polymorphisms in the LDLR 3’ UTR identified in this study Comparison of Allele Frequencies to those in Public Databases The allele frequencies (AFs) of the identified polymorphisms were compared with those reported in public genomic databases, as detailed in Figure 2 and Supplemental Table 2. Except for rs55971831, the AFs of the other ten identified polymorphisms closely matched those observed in East Asian populations within the August 2015 release of the 1000 Genomes Project (1000g2015aug) and the Genome Aggregation Database (gnomAD). The AF for rs55971831 was 0.13 in this cohort, lower than that reported for East Asian populations in both 1000g2015aug and gnomAD. The AFs for rs14158, rs2738466, rs5742911, and rs17249057 were 0.39 in this study. They were slightly lower than the highest recorded AF of 0.41 in East Asian populations, but significantly higher than the AFs observed in American (ranging from 0.21 to 0.29) and African populations (ranging from 0.15 to 0.19). This disparity in AFs suggests a genetic predisposition within the Chinese population for these specific LDLR polymorphisms, underscoring their potential as markers of dyslipidemia in this ethnic group. The AF of rs1433099 was observed to be 0.73 in this study. In contrast, in the 1000g2015aug and gnomAD databases, the AF was reported at 0.79 in American populations, and it ranged between 0.38 and 0.46 in African populations. This indicates that rs1433099 is a common polymorphism across different ethnicities. The AF for rs2738467 was 0.16 in this study, and it was 0.40 to 0.47 in American populations and 0.03 to 0.08 in African populations. This significant variation indicates that the rs2738467 polymorphism exhibits considerable diversity in different populations. The AF of rs7254521 was 0.004 in this study, and this value was 0.003~0.132 in the East Asian population in the public database. However, the AF of rs7254521 was 0.08 in the American population, and approximately 0.15 in the African population. This indicates that rs7254521 has a high ethnic diversity. The polymorphisms rs751672818, rs566918949, and rs568219285 exhibited low AFs in all populations studied, each being less than 0.02. This suggests that these are rare polymorphisms. 198 199 Figure 2 The AFs of the identified polymorphisms in this study and public 200 databases 201 202 203 1000g2015aug: August 2015 release of the 1000 Genomes Project, gnomAD: Genome Aggregation Database, All: All populations, EAS: East Asian, AMR: American, AFR: African. 204 205 Linkage Disequilibrium and Haplotype Analysis 206 The polymorphisms rs14158, rs2738466, rs5742911, and rs17249057, cooccurring in 207 patients, were subjected to linkage disequilibrium analysis. The results, depicted in 208 Figure 3, revealed strong linkage disequilibrium among these polymorphisms (r2=1). 209 The identified haplotypes, GAAT and AGGC, had population allele frequencies of 210 0.614 and 0.386, respectively, in this study cohort. a b c Figure 3 Linkage disequilibrium of LDLR polymorphisms and haplotypes Impact of LDLR Polymorphisms on Serum Lipid Levels at Enrollment The impact of identified polymorphisms on serum lipid levels at enrollment was assessed, with findings summarized in Table 2. Significant associations were observed between polymorphisms rs14158, rs2738466, rs5742911, and rs17249057 and baseline levels of TC and LDL-C (P<0.05). Individuals carrying the A allele of rs14158, the G allele of rs2738466, the G allele of rs5742911, and the C allele of rs17249057 displayed elevated TC and LDL-C levels compared to carriers of alternative alleles. This indicates that such polymorphisms, especially when inherited as a haplotype, could impact LDL-C metabolism. A recent study [40] showed that rs5742911 enhances or

creates a binding site for three miRNAs (miR-3190-5p, miR- 4435, and miR-4717-5p) and disrupts a binding site for miR-1587-5p

, influencing gene expression and potentially contributing to FH, underscoring the significance of the findings in this study. Polymorphism rs1433099 was strongly associated with baseline TC and LDL-C levels (P<0.05). Those who carry the C allele had higher levels of TC and LDL-C. Polymorphism rs55971831 was significantly associated with TG levels (P=0.002), carriers of the A allele exhibiting higher TG levels than those with the C allele. Polymorphism rs568219285 exhibited a significant correlation with baseline TG and TC levels (P<0.05). However, due to its rarity, further validation in a larger cohort is necessary. No significant correlations were observed between polymorphisms rs2738467, rs751672818, rs7254521, or rs566918949 and baseline serum lipid levels. Influence of of LDLR Polymorphisms on Atorvastatin Treatment Efficacy The relationship between LDLR polymorphisms and the relative change in serum lipid levels after atorvastatin therapy was evaluated and was showed in Table 3. Participants carrying the rs2738467 T allele showed a more significant reduction in TC, LDL-C, and HDL-C levels than did those with the C allele (P<0.05). This novel discovery suggests that the rs2738467 T allele might augment the cholesterol- lowering efficacy of atorvastatin. The relative changes in lipid levels in patients with different genotypes at locus rs2738467 after atorvastatin therapy were shown in Figure 4. TC and LDL-C levels reduced 20% in patients carrying the rs2738467 T 13 / 30 allele and 10% in those with the C allele. Although HDL-C levels also decreased in patients with the rs2738467 T allele, the median change was under 5%, with some patients even experiencing an increase in HDL-C levels. This suggests that the rs2738467 T allele may specifically enhance atorvastatin's efficacy in lowering LDL- C levels. The rs1433099 showed a significant correlation with change in HDL-C levels post-atorvastatin treatment (P=0.02). Although patients carrying the rs1433099 C allele presented with greater TC and LDL-C levels at baseline, they showed a greater improvement in HDL-C levels following atorvastatin treatment. The rs7254521 was strongly associated with LDL-C levels post-atorvastatin treatment (P=0.03); however, this observation was limited to only two patients. Verification in larger cohorts is necessary in future studies. No significant correlations were observed between polymorphisms rs14158, rs2738466, rs5742911, rs17249057, rs55971831, rs751672818, rs566918949, or rs568219285 and atorvastatin's efficacy. 260 Table 2 The correlation between LDLR polymorphisms and serum lipid levels at enrollment P rsID gDNA\_coordinate wild/mutant (patients) TG (wild greater) TG (mutant greater) TC TC LDL-C (wild (mutant (wild greater) greater) greater) LDL-C (mutant greater) HDL-C (wild greater) HDL-C (mutant greater) rs1433099 chr19:g.11242658T>C rs14158 chr19:g.11242044G>A rs2738466 chr19:g.11242765A>G rs5742911 chr19:g.11243445A>G rs17249057 chr19:g.11243502T>C rs2738467 chr19:g.11243735C>T rs55971831 chr19:g.11243411C>A rs751672818 chr19:g.11243411delC rs7254521 chr19:g.11243422C>T rs566918949 chr19:g.11243467G>A rs568219285 chr19:g.11242719G>A 21/234 94/161 94/161 94/161 94/161 182/73 188/67 246/9 253/2 253/2 254/1 0.807 0.686 0.686 0.686 0.686 0.467 0.998 0.181 0.17 0.422 0.958 0.193 0.314 0.314 0.314 0.314 0.533 0.002 0.819 0.83 0.578 0.042 0.977 0.023 0.981 0.995 0.005 0.995 0.005 0.995 0.005 0.995 0.005 0.401 0.599 0.386 0.614 0.331 0.669 0.231 0.769 0.692 0.308 0.955 0.045 0.019 0.992 0.008 0.992 0.008 0.992 0.008 0.992 0.008 0.509 0.491 0.264 0.736 0.333 0.667 0.165 0.835 0.803 0.197 0.514 0.486 0.517 0.483 0.844 0.156 0.844 0.156 0.844 0.156 0.844 0.156 0.727 0.273 0.025 0.975 0.658 0.342 0.847 0.153 0.401 0.599 0.061 0.939 Note: Serum lipid levels at enrollment were compared by the Wilcoxon rank sum test. Values in bold are statistically significant (P<0.05). The P values listed in the table represent the null hypothesis, while the remarks in parentheses are indicative of the alternative hypothesis. 15 / 30 267 Table 3 Associations between LDLR polymorphisms and the percentage changes in serum lipid levels after atorvastatin therapy P rsID gDNA\_coordinate wild/mutant (patients) Δ%TG (wild greater) Δ%TG (mutant greater) Δ%TC (wild greater) Δ%TC (mutant greater) Δ%LDL- C (wild greater) Δ%LDL- C (mutant greater) Δ%HDL- C (wild greater) Δ%HDL- C (mutant greater) rs1433099 chr19:g.11242658T>C rs14158 chr19:g.11242044G>A rs2738466 chr19:g.11242765A>G rs5742911 chr19:g.11243445A>G rs17249057 chr19:g.11243502T>C rs2738467 chr19:g.11243735C>T rs55971831 chr19:g.11243411C>A rs751672818 chr19:g.11243411delC rs7254521 chr19:g.11243422C>T rs566918949 chr19:g.11243467G>A rs568219285 chr19:g.11242719G>A 21/234 94/161 94/161 94/161 94/161 182/73 188/67 246/9 253/2 253/2 254/1 0.461 0.539 0.707 0.293 0.707 0.293 0.707 0.293 0.707 0.293 0.309 0.691 0.176 0.824 0.645 0.355 0.5 0.5 0.286 0.714 0.05 0.95 0.571 0.429 0.876 0.124 0.876 0.124 0.876 0.124 0.876 0.124 0.024 0.976 0.712 0.288 0.676 0.324 0.818 0.182 0.238 0.762 0.157 0.843 0.514 0.486 0.851 0.149 0.851 0.149 0.851 0.149 0.851 0.149 0.035 0.965 0.862 0.138 0.337 0.663 0.969 0.031 0.261 0.739 0.876 0.124 0.02 0.639 0.639 0.639 0.639 0.002 0.496 0.984 0.682 0.122 0.942 0.98 0.361 0.361 0.361 0.361 0.998 0.504 0.016 0.318 0.878 0.058 Note: The relative changes in serum lipid levels after atorvastatin therapy were compared by the Wilcoxon rank sum test. Values in bold are statistically significant (P<0.05). The P values listed in the table represent the null hypothesis, while the remarks in parentheses are indicative of the alternative hypothesis. Δ%TG=100\*(TGpostintervention-TGenrollment)/TGenrollment; Δ%TC=100\*(TCpostintervention- TCenrollment/TCenrollment; Δ%LDL-C=100\*(LDL-Cpostintervention-LDL-Cenrollment/LDL-Cenrollment; Δ%HDL-C=100\*(HDL- Cpostintervention-HDL-Cenrollment/HDL-Cenrollment. 16 / 30 a b c Figure 4 The relative changes in lipid levels in patients with different genotypes at locus rs2738467 after atorvastatin therapy a: DeltaTC (%)=100\*(TCpostintervention-TCenrollment)/TCenrollment; b: DeltaLDL-C (%)=100\*(LDL-Cpostintervention-LDL- Cenrollment)/LDL-Cenrollment; c: DeltaHDL-C (%)=100\*(HDL-Cpostintervention-HDL-Cenrollment)/HDL-Cenrollment 17 / 30 Discussion This study not only provided a comprehensive analysis of the correlation between polymorphisms in the LDLR 3’ UTR and baseline serum lipid levels, but also revealed an association between these polymorphisms and the therapeutic efficacy of atorvastatin in a cohort of adult Chinese Han patients with dyslipidemia. The identification of 11 polymorphisms in the LDLR 3’ UTR of these patients underscored the genetic diversity within this population and highlighted the potential of these polymorphisms to serve as biomarkers for the treatment of dyslipidemia. The polymorphisms rs14158, rs2738466, rs5742911, and rs17249057 which were in strong linkage disequilibrium, were significantly correlated with baseline serum lipid levels. Patients with the AGGC haplotype had higher LDL-C levels at baseline. Although an investigation within a southern Chinese population has not established a correlation between polymorphisms rs14158 and rs2738466 and the incidence of coronary heart disease [45], data from a black South African cohort indicated that carriers of the rs14158 A allele have elevated LDL-C levels, increasing the risk for FH [46]. In addition, research conducted in a Spanish population revealed that subjects with hypercholesterolemia harboring the rs14158 A allele and the rs2738466 G allele exhibit a diminished response to the lipid-modulating agent Armolipid Plus, suggesting that these specific SNPs may exacerbate hypercholesterolemia susceptibility [47]. Furthermore, according to a Mexican study, the rs14158 A allele and the rs2738466 G allele were associated with an increased risk of acute coronary syndrome and concomitantly lower HDL-C levels [48]. Additionally, rs5742911 was potentially associated with FH by disrupting interactions with miRNAs and altering gene expression in a recent Dutch study [40]. Collectively, these findings underscore the potential for the rs14158, rs2738466, rs5742911, and rs17249057 polymorphisms to influence cholesterol metabolism in various ways between distinct populations. In this study, polymorphisms rs14158, rs2738466, rs5742911, and rs17249057 were not correlated with the therapeutic efficacy of atorvastatin. This finding was consistent with a study in Brazilian cohorts in which the allelic polymorphism rs14158G had no discernible influence on the therapeutic efficacy of atorvastatin [49]. However, a study in the United States showed that rs5742911 was associated with poor simvastatin response in black patients but not in white patients [50]. The rs2738467 T allele was associated with a more pronounced

reduction in LDL- C
levels
after
atorvastatin
therapy
but
was
not
associated with

baseline lipid levels. This finding suggests a potential role for this polymorphism in improving the efficacy of atorvastatin. This finding supports the precision medicine approach, which emphasizes customizing treatment plans according to individual genetic profiles. The allele frequencies of the identified polymorphisms in this study were consistent with them in East Asian populations as documented in public genomic databases. This reinforces the validity of the findings and suggests a genetic predisposition among the Chinese population to these specific LDLR polymorphisms. The findings in this study have profound implications for population-specific genetic screening and therapeutic interventions. Study strengths and limitations This research presents several strengths, notably its investigation into the effects of LDLR 3’ UTR polymorphisms on lipid levels both pre- and post-atorvastatin therapy in a population of adult Chinese Han individuals with dyslipidemia. The study provides valuable insights into the genetic factors that regulate serum lipids and how the factors impact the efficacy of atorvastatin treatment. This study not only supports 19 / 30 the stratification of potential dyslipidemia cases for targeted screening but also aids in pinpointing individuals most likely to benefit from atorvastatin therapy. As a result, this work lays a foundation for the implementation of personalized, precision medicine in clinical settings. This study still has several limitations. Firstly, focusing exclusively on adult Chinese Han patients with dyslipidemia might restrict the applicability of the findings to other ethnicities or demographics. Secondly, the infrequent presence of certain polymorphisms, like rs568219285, necessitates further exploration in more extensive and varied populations to verify their links to lipid profiles and medication effects. Lastly, while this study concentrated on the relationship between LDLR polymorphisms and lipid levels alterations post-atorvastatin treatment, other contributory factors and underlying mechanisms remain unexamined. Conclusions In conclusion, this investigation has uncovered a significant link between LDLR gene 3’ UTR polymorphisms and lipid levels, as well as their impact on atorvastatin response. These insights open new pathways for advanced studies and clinical applications, highlighting the importance of genetic profiling in tailoring treatment for dyslipidemia. By adopting a personalized approach to therapy, it can enhance treatment precision and effectiveness, ultimately alleviating the cardiovascular disease burden associated with dyslipidemia. List of abbreviations

ASCVD Atherosclerotic cardiovascular disease
LDLR
Low-density lipoprotein
receptor
FH Familial hypercholesterolemia

UTR TG TC LDL-C HDL-C Untranslated regions Triglyceride Total cholesterol Low-density lipoprotein cholesterol High-density lipoprotein cholesterol Supplementary Information Not applicable. Declarations Ethics approval and consent to participate This study was approved by the Ethics Committee of Xiangya Hospital Central South University (ethics number K22144) and The First Hospital of Hebei Medical University (ethics number 20220418), and

all participants provided written informed consent. Consent for publication Written informed consent for publication was obtained from all participants. Availability of data and
materials
The datasets

featured in this article are not openly accessible due to restrictions on the public dissemination of genomic information imposed by

the Institutional Ethics Committee. To access the datasets
, requests
should be
made
to the corresponding authors. Competing interests The authors declare no
competing interests.
Funding
This study
was supported by the

project named Research on Precision Medication for Chronic Diseases Based on Pharmacogenomics (2019YJY0203). Authors' contributions HL.Z.

designed the study
and
carried out all the experiments

. Y.Y. was

primarily responsible for
the
experimental design
. Y.T.
analyzed
the
data and
wrote
the manuscript

. G.Q.Z prepared the figures and tables. N.L.S., S.N.H. and G.

L. designed the research and critically revised the manuscript
. L.
Y.W
., Y.Q.
A

., C.S., M.X.Y., Y.H.Z., Q.L. and Y.W.Z. made modifications to the manuscript. All authors reviewed the manuscript. Acknowledgments

We would like to acknowledge the participants who provided valuable clinical samples for this study

. We express sincere appreciation to the State Key Laboratory of Microbial Resources at the Institute of Microbiology, Chinese Academy of Sciences, for their generous provision of the essential facilities and resources required for this study. References 1. Liu X, Yu S, Mao Z, Li Y, Zhang H, Yang K, et al. Dyslipidemia prevalence, awareness, treatment, control, and risk factors in Chinese rural population: the Henan rural cohort study. Lipids Health Dis. 2018;17(1):119. 2. Lu Y, Zhang H, Lu J, Ding Q, Li X, Wang X, et al. Prevalence of Dyslipidemia and Availability of Lipid-Lowering Medications Among Primary Health Care Settings in China. JAMA Network Open. 2021;4(9). 3. Yang M, Zhang Y, Ren J. Autophagic Regulation of Lipid Homeostasis in Cardiometabolic Syndrome. Frontiers in Cardiovascular Medicine. 2018;5(38). 4. Zhang Y, Whaley-Connell AT, Sowers JR, Ren J. Autophagy as an emerging 22 / 30 target in cardiorenal metabolic disease: From pathophysiology to management. Pharmacol Ther. 2018;191:1-22. 5. Zhang Y, Sowers JR, Ren J. Targeting autophagy in obesity: from pathophysiology to management. Nat Rev Endocrinol. 2018;14(6):356-76. 6. Ren J, Sowers JR, Zhang Y. Metabolic Stress, Autophagy, and Cardiovascular Aging: from Pathophysiology to Therapeutics. Trends Endocrinol Metab. 2018;29(10):699-711. 7. Rogozik J, Główczyńska R, Grabowski M. Genetic backgrounds and diagnosis of familial hypercholesterolemia. Clinical Genetics. 2023;105(1):3-12. 8. Ma LY, Chen WW, Gao RL, Liu LS, Zhu ML, Wang YJ, et al. China cardiovascular diseases report 2018: an updated summary. J Geriatr Cardiol. 2020;17(1):1-8. 9. Ference BA, Ginsberg HN, Graham I, Ray KK, Packard CJ, Bruckert E, et al. Low-density lipoproteins cause atherosclerotic cardiovascular disease. 1. Evidence from genetic, epidemiologic, and clinical studies. A consensus statement from the European Atherosclerosis Society Consensus Panel. Eur Heart J. 2017;38(32):2459-72. 10. Mangione CM, Barry MJ, Nicholson WK, Cabana M, Chelmow D, Coker TR, et al. Statin Use for the Primary Prevention of Cardiovascular Disease in Adults. Jama. 2022;328(8). 11. Ferraro RA, Leucker T, Martin SS, Banach M, Jones SR, Toth PP. 23 / 30 Contemporary Management of Dyslipidemia. Drugs. 2022;82(5):559-76. 12. Li J-J, Zhao S-P, Zhao D, Lu G-P, Peng D-Q, Liu J, et al. 2023 Chinese guideline for lipid management. Front Pharmaco. 2023;14. 13. Polisecki E, Muallem H, Maeda N, Peter I, Robertson M, McMahon AD, et al. Genetic variation at the LDL receptor and HMG-CoA reductase gene loci, lipid levels, statin response, and cardiovascular disease incidence in PROSPER. Atherosclerosis. 2008;200(1):109-14. 14. Weedon MN, Linsel-Nitschke P, Götz A, Erdmann J, Braenne I, Braund P, et al. Lifelong Reduction of LDL-Cholesterol Related to a Common Variant in the LDL-Receptor Gene Decreases the Risk of Coronary Artery Disease—A Mendelian Randomisation Study. PLoS ONE. 2008;3(8). 15. Kathiresan S, Melander O, Anevski D, Guiducci C, Burtt NP, Roos C, et al. Polymorphisms associated with cholesterol and risk of cardiovascular events. N Engl J Med. 2008;358(12):1240-9. 16. Sun XM, Patel DD, Webb JC, Knight BL, Fan LM, Cai HJ, et al. Familial hypercholesterolemia in China. Identification of mutations in the LDL-receptor gene that result in a receptor-negative phenotype. Arterioscler Thromb. 1994;14(1):85-94. 17. Pimstone SN, Sun XM, du Souich C, Frohlich JJ, Hayden MR, Soutar AK. Phenotypic variation in heterozygous familial hypercholesterolemia: a comparison of Chinese patients with the same or similar mutations in the LDL 24 / 30 receptor gene in China or Canada. Arterioscler Thromb Vasc Biol. 1998;18(2):309-15. 18. Wang D, Wu B, Li Y, Heng W, Zhong H, Mu Y, et al. A Chinese homozygote of familial hypercholesterolemia: identification of a novel C263R mutation in the LDL receptor gene. J Hum Genet. 2001;46(3):152-4. 19. Punzalan FE, Sy RG, Santos RS, Cutiongco EM, Gosiengfiao S, Fadriguilan E, et al. Low density lipoprotein--receptor (LDL-R) gene mutations among Filipinos with familial hypercholesterolemia. J Atheroscler Thromb. 2005;12(5):276-83. 20. Xie L, Gong QH, Xie ZG, Liang ZM, Hu ZM, Xia K, et al. Two novel mutations of the LDL receptor gene associated with familial hypercholesterolemia in a Chinese family. Chin Med J (Engl). 2007;120(19):1694-9. 21. Cheng X, Ding J, Zheng F, Zhou X, Xiong C. Two mutations in LDLR gene were found in two Chinese families with familial hypercholesterolemia. Mol Biol Rep. 2009;36(8):2053-7. 22. Wang L, Lin J, Liu S, Cao S, Liu J, Yong Q, et al. Mutations in the LDL receptor gene in four Chinese homozygous familial hypercholesterolemia phenotype patients. Nutr Metab Cardiovasc Dis. 2009;19(6):391-400. 23. De Castro‐Orós I, Pampín S, Bolado‐Carrancio A, De Cubas A, Palacios L, Plana N, et al. Functional analysis of LDLR promoter and 5′ UTR mutations in subjects with clinical diagnosis of familial hypercholesterolemia. Hum Mutat. 2011;32(8):868-72. 25 / 30 24. Yao RE, Wang J, Geng J, Zheng Z, Yu T, Yu Y, et al. Identification of LDLR mutations in two Chinese pedigrees with familial hypercholesterolemia. J Pediatr Endocrinol Metab. 2012;25(7-8):769-73. 25. Li H, Zhang Y, Wei X, Peng Y, Yang P, Tan H, et al. Rare intracranial cholesterol deposition and a homozygous mutation of LDLR in a familial hypercholesterolemia patient. Gene. 2015;569(2):313-7. 26. Santos PC, Pereira AC. Type of LDLR mutation and the pharmacogenetics of familial hypercholesterolemia treatment. Pharmacogenomics. 2015;16(15):1743- 50. 27. Ohta N, Hori M, Takahashi A, Ogura M, Makino H, Tamanaha T, et al. Proprotein convertase subtilisin/kexin 9 V4I variant with LDLR mutations modifies the phenotype of familial hypercholesterolemia. J Clin Lipidol. 2016;10(3):547-55. 28. Climent E, Pérez-Calahorra S, Marco-Benedí V, Plana N, Sánchez R, Ros E, et al. Effect of LDL cholesterol, statins and presence of mutations on the prevalence of type 2 diabetes in heterozygous familial hypercholesterolemia. Sci Rep. 2017;7(1):5596. 29. Shu H, Chi J, Li J, Zhang W, Lv W, Wang J, et al. A novel indel variant in LDLR responsible for familial hypercholesterolemia in a Chinese family. PLoS One. 2017;12(12). 30. Girona J, Rodríguez-Borjabad C, Ibarretxe D, Heras M, Amigo N, Feliu A, et 26 / 30 al. Plasma inducible degrader of the LDLR, soluble low-density lipoprotein receptor, and proprotein convertase subtilisin/kexin type 9 levels as potential biomarkers of familial hypercholesterolemia in children. J Clin Lipidol. 2018;12(1):211-8. 31. Hoffman S, Adeli K. LDL Receptor Gene-Ablated Hamsters: A Rodent Model of Familial Hypercholesterolemia with Dominant Inheritance and Diet-Induced Coronary Atherosclerosis. EBioMedicine. 2018;28:17-8. 32. Rodríguez-Nóvoa S, Rodríguez-Jiménez C, Alonso C, Rodriguez-Laguna L, Gordo G, Martinez-Glez V, et al. Familial hypercholesterolemia: A single- nucleotide variant (SNV) in mosaic at the low density lipoprotein receptor (LDLR). Atherosclerosis. 2020;311:37-43. 33. Zhimin W, Hui W, Fengtao J, Wenjuan S, Yongrong L. Clinical and serum lipid profiles and LDLR genetic analysis of xanthelasma palpebrarum with nonfamilial hypercholesterolemia. J Cosmet Dermatol. 2020;19(11):3096-9. 34. Doi T, Hori M, Harada‐Shiba M, Kataoka Y, Onozuka D, Nishimura K, et al. Patients With LDLR and PCSK9 Gene Variants Experienced Higher Incidence of Cardiovascular Outcomes in Heterozygous Familial Hypercholesterolemia. J Am Heart Assoc. 2021;10(4). 35. Hu H, Chen R, Hu Y, Wang J, Lin S, Chen X. The LDLR c.501C>A is a disease- causing variant in familial hypercholesterolemia. Lipids Health Dis. 2021;20(1):101. 27 / 30 36. Meshkov A, Ershova A, Kiseleva A, Zotova E, Sotnikova E, Petukhova A, et al. The LDLR, APOB, and PCSK9 Variants of Index Patients with Familial Hypercholesterolemia in Russia. Genes. 2021;12(1). 37. Roy G, Couture P, Genest J, Ruel I, Baass A, Bergeron J, et al. Influence of the LDL-Receptor Genotype on Statin Response in Heterozygous Familial Hypercholesterolemia: Insights From the Canadian FH Registry. Can J Cardiol. 2022;38(3):311-9. 38. Lin S, Hu T, Wang K, Wang J, Zhu Y, Chen X. In vitro assessment of the pathogenicity of the LDLR c.2160delC variant in familial hypercholesterolemia. Lipids Health Dis. 2023;22(1):77. 39. Lv X, Wang C, Liu L, Yin G, Zhang W, Abdu FA, et al. Screening and verifying the mutations in the LDLR and APOB genes in a Chinese family with familial hypercholesterolemia. Lipids Health Dis. 2023;22(1):175. 40. de Freitas RCC, Bortolin RH, Borges JB, de Oliveira VF, Dagli-Hernandez C, Marçal EdSR, et al. LDLR and PCSK9 3´UTR variants and their putative effects on microRNA molecular interactions in familial hypercholesterolemia: a computational approach. Mol Biol Rep. 2023;50:9165–77. 41. Bolger AM, Lohse M, Usadel B. Trimmomatic: a flexible trimmer for Illumina sequence data. Bioinformatics. 2014;30(15):2114-20. 42. Li H, Durbin R. Fast and accurate long-read alignment with Burrows- Wheeler transform. Bioinformatics. 2010;26(5):589-95. 28 / 30 43. McKenna A, Hanna M, Banks E, Sivachenko A, Cibulskis K, Kernytsky A, et al. The Genome Analysis Toolkit: a MapReduce framework for analyzing next- generation DNA sequencing data. Genome Res. 2010;20(9):1297-303. 44. Barrett JC. Haploview: Visualization and analysis of SNP genotype data. Cold Spring Harb Protoc. 2009;4(10). 45. Chen W, Wang S, Ma Y, Zhou Y, Liu H, Strnad P, et al. Analysis of polymorphisms in the 3' untranslated region of the LDL receptor gene and their effect on plasma cholesterol levels and drug response. Int J Mol Med. 2008;21(3):345-53. 46. van Zyl T, Jerling JC, Conradie KR, Feskens EJM. Common and rare single nucleotide polymorphisms in the LDLR gene are present in a black South African population and associate with low-density lipoprotein cholesterol levels. J Hum Genet. 2013;59(2):88-94. 47. Vinci MC, De Castro-Orós I, Solà R, Valls RM, Brea A, Mozas P, et al. Genetic Variants of LDLR and PCSK9 Associated with Variations in Response to Antihypercholesterolemic Effects of Armolipid Plus with Berberine. Plos One. 2016;11(3). 48. Vargas-Alarcon G, Perez-Mendez O, Ramirez-Bello J, Posadas-Sanchez R, Gonzalez-Pacheco H, Escobedo G, et al. The c.\*52 A/G and c.\*773 A/G Genetic Variants in the UTR'3 of the LDLR Gene Are Associated with the Risk of Acute Coronary Syndrome and Lower Plasma HDL-Cholesterol Concentration. 29 / 30 Biomolecules. 2020;10(10). 49. Zambrano T, Hirata MH, Cerda Á, Dorea EL, Pinto GA, Gusukuma MC, et al. Impact of 3'UTR genetic variants in PCSK9 and LDLR genes on plasma lipid traits and response to atorvastatin in Brazilian subjects: a pilot study. Int J Clin Exp Med. 2015;8(4):5978-88. 50. Mangravite LM, Medina MW, Cui J, Pressman S, Smith JD, Rieder MJ, et al. Combined influence of LDLR and HMGCR sequence variation on lipid-lowering response to simvastatin. Arterioscler Thromb Vasc Biol. 2010;30(7):1485-92. 30 / 30 1 2 3 4 5 6 7 8 9 10 11 12 13 14 15 16 17 18 19 20 21 22 23 24 25 26 27 28 29 30 31 32 33 34 35 36 37 38 39 40 41 42 43 44 45 46 47 48 49 50 51 52 53 54 55 56 57 58 59 60 61 62 63 64 65 66 67 68 69 70 71 72 73 74 75 76 77 78 79 80 81 82 83 84 85 86 87 88 89 90 91 92 93 94 95 96 97 98 99 100 101 102 103 104 105 106 107 108 109 110 111 112 113 114 115 116 117 118 119 120 121 122 123 124 125 126 127 128 129 130 131 132 133 134 135 136 137 138 139 140 141 142 143 144 145 146 147 148 149 150 151 152 153 154 155 156 157 158 159 160 161 162 163 164 165 166 167 168 169 170 171 172 173 174 175 176 177 178 179 180 181 182 183 184 185 186 187 188 189 190 191 192 193 194 195 196 197 211 212 213 214 215 216 217 218 219 220 221 222 223 224 225 226 227 228 229 230 231 232 233 234 235 236 237 238 239 240 241 242 243 244 245 246 247 248 249 250 251 252 253 254 255 256 257 258 259 261 262 263 264 265 266 268 269 270 271 272 273 274 275 276 277 278 279 280 281 282 283 284 285 286 287 288 289 290 291 292 293 294 295 296 297 298 299 300 301 302 303 304 305 306 307 308 309 310 311 312 313 314 315 316 317 318 319 320 321 322 323 324 325 326 327 328 329 330 331 332 333 334 335 336 337 338 339 340 341 342 343 344 345 346 347 348 349 350 351 352 353 354 355 356 357 358 359 360 361 362 363 364 365 366 367 368 369 370 371 372 373 374 375 376 377 378 379 380 381 382 383 384 385 386 387 388 389 390 391 392 393 394 395 396 397 398 399 400 401 402 403 404 405 406 407 408 409 410 411 412 413 414 415 416 417 418 419 420 421 422 423 424 425 426 427 428 429 430 431 432 433 434 435 436 437 438 439 440 441 442 443 444 445 446 447 448 449 450 451 452 453 454 455 456 457 458 459 460 461 462 463 464 465 466 467 468 469 470 471 472 473 474 475 476 477 478 479 480 481 482 483 484 485 486 487 488 489 490 491 492 493 494 495 496 497 498 499 500 501 502 503 504 505 506 507 508 509 510 511 512 513 514 515 516 517 518 519 520 521 522 523 524 525 526 527 528 529 530 531 532 533 534 535 536 537 538 539 540 541 542 543 544 545 546 1 / 30 2 / 30 4 / 30 5 / 30 7 / 30 8 / 30 9 / 30 10 / 30 11 / 30 12 / 30 14 / 30 18 / 30 20 / 30 21 / 30

Loading, please wait...
